# Current Advances in CAR T Cell Therapy for Malignant Mesothelioma

**DOI:** 10.33696/immunology.2.042

**Published:** 2020

**Authors:** Astero Klampatsa, Steven M. Albelda

**Affiliations:** 1 Division of Cancer Therapeutics, The Institute of Cancer Research, London, UK; 2 Division of Pulmonary, Allergy and Critical Care, Perelman School of Medicine, University of Pennsylvania, Philadelphia, Pennsylvania, USA

**Keywords:** Chimeric antigen receptor T cells, Mesothelioma, Adoptive cell transfer, Solid tumor, Tumor microenvironment

## Abstract

Malignant mesothelioma is a relatively rare malignancy arising in the body’s serosal surfaces, with malignant pleural mesothelioma (MPM) being the most common type. It is characterized by local spread within the thorax, poor prognosis and resistance to treatment. The development of various immunotherapeutic options has provided a new way- and hope- in treating cancer patients. Chimeric antigen receptor (CAR) T cell therapy has been proven very successful in treating hematological cancers, like leukemias and lymphomas, and its use is now being tested in solid tumors. CARs that recognize and bind to a specific tumor-associated antigen on the tumor’s cell surface, are engineered and transduced into T cells. Interaction of the CAR T cell with the tumor then results in T cell activation and subsequent tumor cell lysis. In this review, we provide a current update on our previous comprehensive study summarizing the CAR T cell preclinical studies and clinical trials in MM, and discuss the future perspectives of CAR T cell therapy in this disease.

## Introduction

Malignant mesothelioma (MM) is an incurable primary tumor of the body’s serosal surfaces: the pleura, peritoneum, pericardium and the tunica vaginalis (in men). It is causally linked to occupational or environmental exposure to asbestos [1-3], a natural mineral that has been recognised as a carcinogen and its mining and use has been subsequently banned or reduced. Despite the severe health implications in using asbestos and the stringent regulations on its use imposed, the asbestos industry is still present in many countries worldwide. Given the long latency period from exposure to diagnosis [4], the incidence of MM is still increasing in some countries. The UK is currently in the midst of a ‘mesothelioma epidemic’ with the number of deaths having increased from 153 per annum in 1968 to 2,046 in 2005 and currently affecting around 3,000 people annually [5]. The incidence of MM in the United States peaked in around 2000 and it is currently estimated to be around 3300 cases per year [6].

Malignant pleural mesothelioma (MPM) arises in the parietal pleura, spreads contiguously to invade local structures and can pass through the diaphragm into the abdominal cavity. MPM accounts for around 65% to 70% of all mesotheliomas, while those arising in other mesothelial membranes (peritoneal around 30%; pericardial 1-2%) are much less common [7]. Histopathologically, there are three main subtypes of mesothelioma: epithelioid, sarcomatoid and biphasic [4]. The disease is characterised by profound resistance to therapy and poor prognosis.

There is currently no known cure for MM, therefore any treatment aims to improve quality of life and extend survival. Standard treatments may involve a combination of surgery, chemotherapy and radiotherapy (trimodality therapy). Radical surgery aiming for complete tumor resection, either by sacrificing the neighboring/affected lung (extrapleural pneumonectomy; EPP) or by sparing it (pleurectomy/decortication; P/D), can be beneficial to a small cohort of selected patients [8,9]. The mainstay of treatment in MPM, whether after surgery or in unresectable tumors, has been chemotherapy. Although the only FDA-approved frontline therapy is pemetrexed with platinum (cisplatin or carboplatin) [10], other chemotherapeutic agents including gemcitabine and vinorelbine have also been used [11]. Radiotherapy is being used to the surgical wound to prevent tumor seeding (prophylactic radiotherapy) after surgery, or in the palliative setting [12]. To date, the outcomes of multimodality therapy regimens have been disappointing with modest survival benefit of 9-17 months, emphasizing the need for more effective treatments [13,14].

## Chimeric Antigen Receptors (CARs)

**Figure 1:**
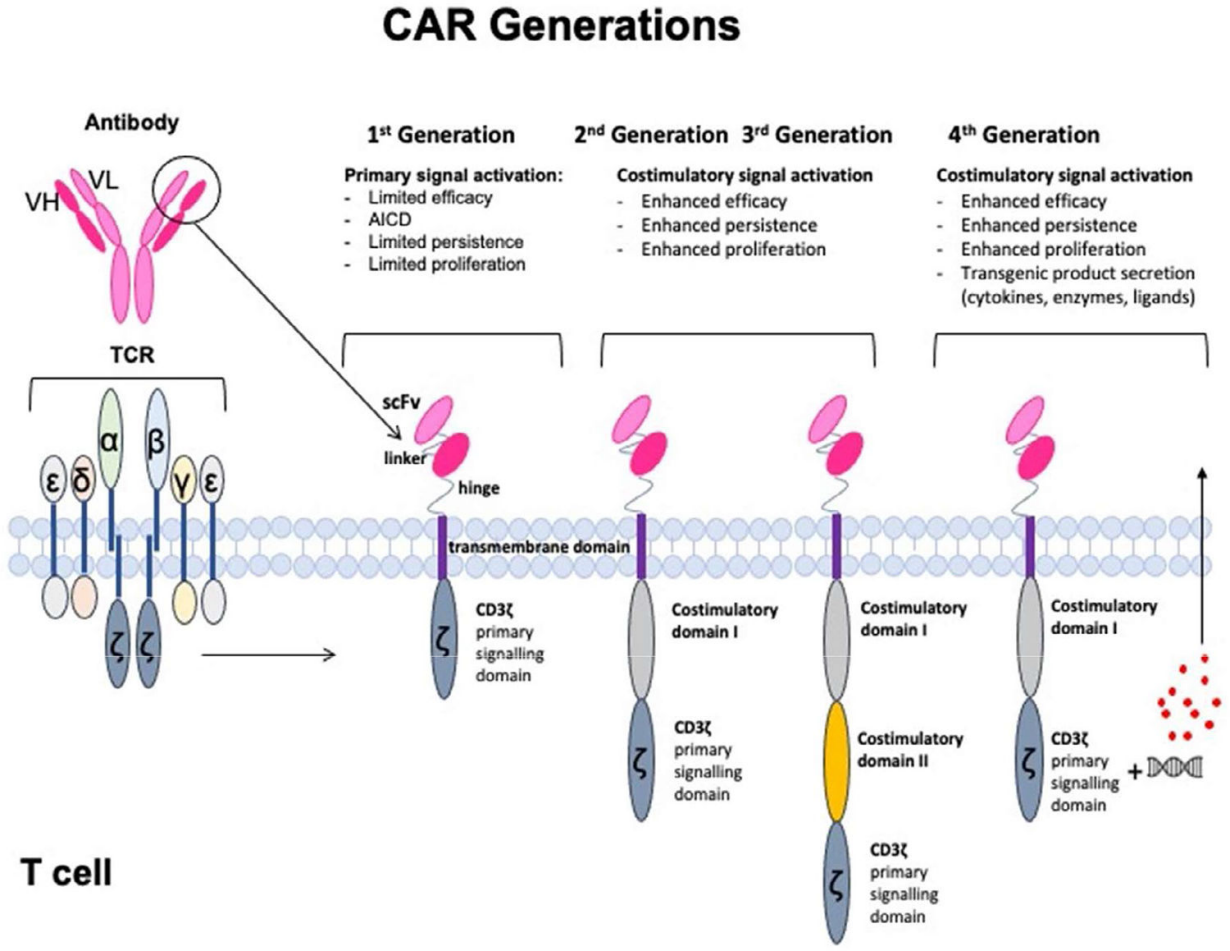
Generations of chimeric antigen receptor (CAR). The single chain scFv- derived from the heavy (VH) and light (VL) antigen-binding domains of antibodies- is fused through a hinge and a transmembrane domain to CD3ζ, the primary signalling domain from the T-cell receptor (TCR) complex; this is the first-generation CAR. Additional intracellular domains (such as CD28 and 41BB) added for costimulatory signals, to yield second- and third- generation CARs. In addition to costimulatory signals, the fourth generation CAR (also referred to as “TRUCK”) incorporates transgenic expression of stimulatory molecules (cytokines, enzymes, or ligands).

Chimeric antigen receptors (CARs) are engineered T cell receptor-like molecules whose antibody-based extracellular targeting moieties provide exquisite specificity to tumor associated antigens (TAAs) and result in T cell activation in a predictable fashion. CARs can bind directly to a variety cell surface targets (including proteins, lipids and carbohydrates), independently of MHC presentation of the antigen and provide thus a wide targeting repertoire [15]. Intracellularly, the CAR’s main component is the CD3ζ activating signaling domain, with additional costimulatory domains fused that provide lasting T cell activation and survival [15,16]. The extra- and intra-cellular domains are fused together with appropriate spacer and transmembrane molecules. The multiple design options resulted in “generations” of CARs. “First generation” CARs consist of an extracellular domain that binds to the tumor antigen via a single-chain variable antibody fragment (scFv) that is fused to a CD3ζ intracellular activating domain [17]. “Second generation” CARs have ccommonly incorporated on co-stimulatory domain, like CD28 and 4-1BB, to the primary signaling domain CD3ζ [17]. CARs with combined co-stimulatory domains are termed “third generation”. The addition of one or more co-stimulatory domains led to enhanced overall CAR T cell effector function, increased T-cell proliferation and persistence, delayed apoptosis, and markedly improved anti-tumor efficacy *in vivo* [18,19]. “Fourth generation” CARs, termed TRUCK (T cells Redirected for Universal Cytokine-mediated Killing) CARs have the ability to release transgenic ‘payload’ (cytokines, enzymes, co-stimulatory ligands) upon CAR T cell activation [20]. The four generations of CAR designs are drawn in Figure 1.

Other CAR T cell designs include: a) Two co-expressed CARs so that two TAAs on a tumor cell can be synchronously recognized [21], b) A bispecific CAR (TanCAR) that transmits the activating signal upon engagement of either antigen 1 or antigen 2 or both [22], and c) an inhibitory CAR (iCAR) that provides a blocking signal upon antigen binding [23].

CARs can be inserted into autologous T cells using viral (lentiviral or retroviral) or non-viral (transposon) gene transfer systems to achieve permanent CAR expression or using messenger RNA (mRNA) electroporation to achieve transient expression for toxicity assessment [24,25]. Following transduction, the produced CAR T cells are expanded *ex vivo* in specialized gene transfer facilities and re-infused to the patient, either systemically or regionally, as a therapeutic intervention. CARs targeting the B-cell antigen CD19, have shown dramatic results in clinical trials for a number of hematologic B cell malignancies (ALL, CLL, and lymphoma) [26-28], and have provided the “proof of principal” rationale for CAR T cell development in a variety of solid tumors, including MPM [18]. A requirement for successful CAR T cell therapy, however, is a specific and highly expressed candidate TAA. In MPM, two such candidate target TAAs are currently being investigated in clinical trials: mesothelin, which is overexpressed on the tumor cells, and fibroblast activation protein (FAP) that is overexpressed on tumor stromal cells [18].

## Mesothelin-targeting CARs

Mesothelin is a cell-surface glycoprotein that is expressed at low levels on normal tissues whereas it is overexpressed in the majority of MPM, as well as in lung, pancreatic, and ovarian carcinomas [29]. Mesothelin is an especially appealing target antigen since several preclinical and clinical studies found that it was involved in the malignant transformation of tumors and had a clear association with tumor aggressiveness, which led to local invasion and eventual metastasis [30,31]. Given its overexpression in MPM, versus limited expression on normal mesothelial cells of the pleura, pericardium, peritoneum, and tunica vaginalis, mesothelin-targeting CARs have been extensively studied in the preclinical and clinical setting.

Based on potent anti-tumor effects observed in preclinical studies using mRNA electroporation [32], an initial study focusing on toxicity assessment was conducted (NCT01355965) at the University of Pennsylvania, US, using T cells that only transiently expressed the second-generation murine anti-mesothelin CAR that contained the CD3ζ and 41BB signalling domains [24,33]. No patient in this phase I safety trial demonstrated “on target, off tumor” toxicity (pleuritis, peritonitis, pericarditis) from CAR mesothelin T cell infusion; however, no evident clinical responses were attained [18,24]. Interestingly, an immediate serious anaphylactic reaction was noted in one patient during a delayed mesothelin CAR T cell infusion that was attributed to the immunogenicity of the murine SS1 scFv used in the CAR construct [33]. Given safety confirmation with transient CAR mesothelin expression, a second phase 1 trial (NCT02159716) was conducted in 15 patients with mesothelioma, ovarian cancer and pancreatic cancer using a lentiviral transduction vector expressing the same murine-based anti-mesothelin second generation CAR [34]. In this trial, two different doses of T cells were administered and cyclophosphamide was added as a lymphodepletion agent in some cohorts. The mesothelin CAR T cells were well tolerated and CARexpressing cells in the blood could be detected using qPCR for about 30 days. Cyclophosphamide pre-treatment enhanced CART-meso expansion but did not improve persistence beyond 28 days [34]. Unfortunately, limited clinical activity was reported, with best overall response being stable disease in 11/15 patients [34]. A third trial has been initiated at the University of Pennsylvania using a more active, fully human anti-mesothelin CAR, with addition of cyclophosphamide and using different routes of administration (intravenous and intrapleural respectively) aiming in overall enhanced CAR T cell persistence and efficacy (NCT03054298).

Investigators at the Memorial Sloan Kettering are also conducting mesothelin-targeting CAR T cell trial for the treatment of malignant pleural disease, including MPM (NCT02414269). Their approach is based on preclinical studies in an orthotopic MPM mouse model showing potent and long-lasting antitumor efficacy of intrapleurally-administered mesothelin CAR T-cell therapy [35]. This phase I/II clinical trial uses a CAR with a human-derived anti-mesothelin scFv and a CD3Z/CD28 signalling domain transduced using a retroviral vector and is being administered intrapleurally in patients with primary or secondary pleural malignancies, with MPM being the primary target population. A subset of the MPM patient cohort is also subsequently administered Pembrolizumab, a PD-1 checkpoint inhibitor, to test whether it prolonged the activity of CAR T cell therapy. Preliminary results from 27 patients (25 of whom had MPM) were presented at the 2019 American Society of Clinical Oncology (ASCO) meeting and showed that, of the patients who received cyclophosphamide, CAR-T cell therapy, and who had at least 3 doses of Pembrolizumab, 63% of patients achieved either a partial response or a complete response [36]. Additionally, in these patients, the CAR T cells persisted in the pleural fluid for up to 42 weeks [36]. This promising trial is currently recruiting. A summary of all mesothelin-targeting CAR T cell clinical trials is summarized in Table 1 below.

## Other CARs Targeting Tumor Antigens in MPM Being Studied Preclinically

### Pan-ErbB ‘T4’ CAR

Klampatsa et al. investigated the efficiency of a CAR targeting the four members of the ErbB family (EGFR, HER2, ErbB3, ErbB4) in MPM [37]. They firstly demonstrated expression of EGFR and ErbB4 in a cohort of MPM histological samples, which provided the rationale for testing this CAR in MPM [37]. To redirect T-cell specificity against the ErbB family, they engineered a second generation CAR named T1E28Z [37]. The CAR is co-expressed with a chimeric cytokine receptor named 4ab that delivers an interleukin (IL)-2/IL-15 signal upon binding of IL-4, thereby enabling the selective enrichment of CAR T-cells during ex *vivo* expansion [37]. The study used MPM patients’ blood and showed that successful transduction and enrichment of CAR T-cells was achieved in all patients, either at diagnosis or following chemotherapy [18]. Functionality of the expanded cells was indicated both *in vitro* and *in vivo*. These data provided support for clinical evaluation of intra-cavitary T4 immunotherapy in MPM patients in a Phase 1 clinical trial, subject to funding acquisition.

### 5T4 CAR

The oncofetal cell surface glycoprotein, 5T4, is overexpressed in numerous malignancies, including testicular, breast and colon cancer, while its expression in normal tissues is restricted to specialized epithelial cells [38], making it another promising immunotherapy target. Recently, it was reported that 5T4 was also expressed in a very high percentage of MPM cell lines and biopsies and pleural fluid samples [39]. Interestingly, 5T4-specific CD8^+^ T-cells were able to kill four out of six HLA-A2+ MPM cell lines but not an HLA-A2– cell line, demonstrating immune recognition of MPM-associated 5T4 antigen at the effector T-cell level [39]. Given that a 5T4 CAR has recently been generated and shown to efficiently kill 5T4-expressing nasopharyngeal carcinoma cells *in vitro* [40], 5T4 CARs represent a promising therapeutic strategy for MPM.

### Chondroitin sulfate proteoglycan CARs

The cell surface proteoglycan chondroitin sulphate proteoglycan 4 (CSPG4) has been found to be overexpressed MPM where it has been reported that CSPG4 was overexpressed in 6 out of 8 MPM cell lines, and in 25 out of 41 MPM biopsies [41]. In 2014, investigators from the National Cancer Institute in the United States constructed four 2nd generation CARs, each from a different murine monoclonal antibody, linked to the CD28 co-stimulatory domain and the intracellular T cell receptor signalling chain CD3ζ [24,42]. Donor T cells transduced with these CARs demonstrated cytokine release and cytolytic function when co-cultured with several tumor cell lines, including MPM [42]. The authors concluded that CSPG4 is an attractive target for CAR T-cell therapy, yet some issues were raised about lowlevel expression of this protein in normal small bowel samples [42].

### MET CAR

MET is a single pass tyrosine kinase receptor, normally expressed by cells of epithelial origin, but abnormally activated and overexpressed in a variety of cancers, including MM [43,44]. Thayaparan et al. engineered MET-specific CARs in which a CD28+CD3ζ endodomain was fused to one of three peptides from the N and K1 domains of hepatocyte growth factor (HGF), the minimum MET binding element present in HGF [45]. The developed constructs were demonstrated to show cytotoxicity against MET-expressing MPM cell lines *in vitro* and in an orthotopic (abdominal) tumor model in *vivo,* with no adverse effects.

### CARs Targeting Non-Tumor Antigens in MPM

In addition to tumor cell-specific antigens, it has been proposed that CARs that target essential components of the tumor-associated stroma, such as fibroblasts or endothelial cells, might also be valuable to enhance anti-tumor activity [18]. There are multiple potential advantages to this approach: 1) stromal cells are more genetically stable and less likely to lose antigen expression, 2) attacking the stromal components may alter the tumor microenvironment to improve standard chemotherapeutic or ACT efficacy, and 3) it could be used on multiple different tumor types [18]. Two proposed stromal candidates are fibroblast activation protein (FAP) and vascular endothelial growth factor receptor 2 (VEGFR2), of which FAP is being explored in MPM.

**Figure 2:**
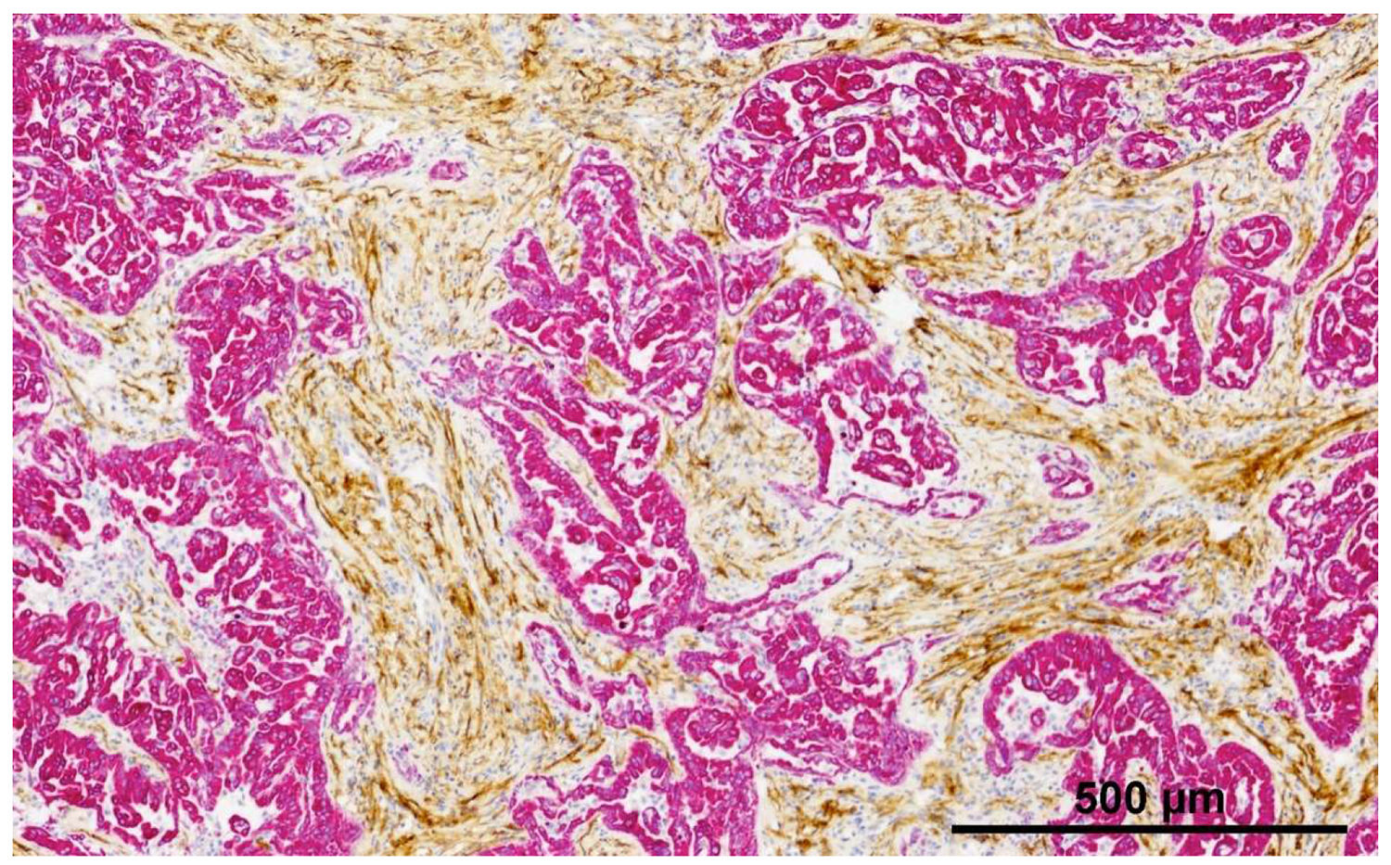
Fibroblast Activating Protein (FAP) immunohistochemical staining in MPM. Histological slide of an MPM tumor shows dual staining of tumor cells (red; cytokeratin) and cancer-associated fibroblasts staining (brown; FAP).

FAP is a transmembrane serine protease, which is highly expressed in the cancer-associated stromal cells (CASCs) of virtually all epithelial cancers with low expression on normal cells [46]. FAP is overexpressed in all three major MPM subtypes including epithelioid, sarcomatoid, and biphasic [47]. Figure 2 shows an example of FAP staining in two mesothelioma tumors.

Preclinical studies have demonstrated that CAR T cells targeted to murine FAP have anti-tumor efficacy in MPM models with minimal toxicity [46]. An anti-human directed FAP CAR with the CD3ζ and CD28 signaling domains was produced at the University of Zurich and shown to induce killing of tumor cells expressing human FAP [47]. Based on these preclinical studies, this group has initiated a phase I clinical trial to evaluate the safety of administering FAP-redirected T cells intrapleurally to patients with MPM (NCT01722149) [48]. Preliminary results presented at the European Society of Molecular Oncology (ESMO) Congress in Autumn 2019 showed that, in 3 MPM patients treated, there was good tolerance of treatment and some persistence of CAR-T cells was seen. With a median follow-up of 18 months, 2 out of 3 patients were alive [49].

## The Future: CAR Augmentation Strategies to Overcome the Challenges of MPM TME

To date, the success of CAR T cells seen in hematologic malignancies has not yet been reproduced in solid tumors and successful MPM treatment with CAR T cells will doubtlessly be challenging. This will be due to multiple mechanisms that include insufficient T cell trafficking to the tumor, CAR T cell suppression due to soluble mediators (like adenosine, TGFβ, and prostaglandin E2), upregulation of checkpoint inhibitors (like PD1) on the CARs, and CAR suppression due to intrinsic inhibitory T cell programs [50]. In addition, TAA expression heterogeneity and immune escape could also be potential issues.

Many groups are developing approaches aiming to overcome these hurdles. Combination of CAR T cells with systemic drugs that affect immune function in general, such as checkpoint blockade using antibodies [51], inhibitors of immunosuppressive agents, like indoleamine 2-3 dioxygenase (IDO) [52], adenosine, PGE2, or immunosuppressive cell types (like CD4 T-regulatory cells) have been explored. The Adusumilli group has investigated the effect of PD-1/PD-L1 mediated T-cell exhaustion on mesothelin CAR T cells using an orthotopic mouse model of MPM [53]. Their study demonstrated that, following repeated antigen exposure, CAR T cells become exhausted and non-functional [54]. This was attributed to PD-1 upregulation on CAR T cells, with corresponding tumor cell upregulation of PD-L1 and PD-L2 in response to effector cytokines exposure, and they demonstrated that exhausted CAR T cells can be rescued by PD-1 checkpoint blockade [54]. CARs can also be combined with other types of immunotherapy such as oncolytic viruses or whole-cell vaccines.

The ability to genetically manipulate CARs allows the possibility of generating improved CARs by inserting or removing specific genes, thus adding an additional level of opportunity and excitement. Aiming to improve CAR T cell trafficking, Moon et al. explored the efficacy of genetically enhancing a matched chemokine receptor (CCR2b) that is expressed on mesothelin CAR T cells in response to measuring elevated levels of the corresponding tumor-secreted chemokine (CCL2) by the MPM cells [55]. This preclinical *in vivo* study demonstrated that transduction of CCR2b onto mesothelin-targeting CAR T cells was able to significantly enhance tumor localization, infiltration, and eradication [55]. Liu et al. successfully demonstrated the utility of combining CAR T cells with a PD1/CD28 chimeric switch-receptor to overcome the suppressive effects of this inhibitory regulator [56]. This switch-receptor fuses an extracellular PD-1 domain to a cytoplasmic CD28 domain, which stimulates the T-cell upon binding to the PD-L1 ligand, thereby activating, rather than inhibiting, T-cell effector function [56]. Administration of PD1CD28 resulted in significantly increased levels of T-cell infiltration, cytokine secretion, and lytic function with long-term statistical reductions in tumor volume [56].

Other examples include: TGFβ blockade expressing a decoy receptor [57], inhibition of adenosine and PGE2 immunosuppressive effects by inserting a small peptide that prevents activation of protein kinase A [58], delivery of activating cytokines (like IL-12) [20] and improved CAR T cell function by using cytoplasmic domains derived from natural killer cells [59]. Many other approaches are actively being pursued.

## Conclusion

The exciting successes seen in hematologic malignancies have prompted development of CAR T-cell therapy for solid tumors, such as MPM. Mesothelioma has two potential advantages. First, a relatively safe and specific TAA (mesothelin) has been identified. Second, mesothelioma may provide an opportunity to use local therapy by intrapleural or intratumoral injection. However, although clinical trials of CARs for use in mesothelioma are underway, clear success of CAR T cells in any solid tumor has not yet been achieved. Accordingly, the results of these trials and knowledge gained from CAR T cell trials in other solid tumors will need to be used iteratively to improve the next series of trials, hopefully eventually leading to adoptive T cell transfer as an important part of the MPM therapeutic armamentarium.

## Figures and Tables

**Table 1:** Clinical trials of mesothelin-targeted CAR T cells in MPM.

Clinical Trial	Phase	Setting	Intervention	Status	Location
NCT02580747	I	Malignant Mesothelioma, Pancreatic Cancer, Ovarian Tumour, Triple Negative Breast Cancer, Endometrial Cancer, Other Mesothelin Positive Tumours	Anti-meso-CAR T cells	Unknown	Chinese PLA General Hospital, Beijing, China
NCT03747965	I	Solid Tumour, Adult	Mesothelin-directed CAR-T cells	Recruiting	Chinese PLA General Hospital, Beijing, China
NCT03054298	I	Lung Adenocarcinoma, Ovarian Cancer, Peritoneal Carcinoma, Fallopian Tube Cancer, Mesotheliomas Pleural, Mesothelioma Peritoneum	huCART-meso cells	Active, not recruiting	University of Pennsylvania, Philadelphia, PA, USA
NCT02414269	I/II	Malignant Pleural Disease, Mesothelioma, Metastases, Lung Cancer, Breast Cancer	MSLN iCasp9M28z T cell infusions Drug: cyclophosphamide Drug: pembrolizumab	Recruiting	Memorial Sloan Kettering, New York City, NY, USA
NCT02159716	I	Metastatic Pancreatic (Ductal) Adenocarcinoma, Epithelial Ovarian Cancer, Malignant Epithelial Pleural Mesothelioma	CART-meso	Completed	University of Pennsylvania, Philadelphia, PA, USA
NCT01583686	I/II	Cervical Cancer, Pancreatic Cancer, Ovarian Cancer, Mesothelioma, Lung Cancer	Anti-mesothelin chimeric T cell receptor (CAR) transduced peripheral blood lymphocytes (PBL) Drug: Cyclophosphamide Drug: Aldesleukin Drug: Fludarabine	Terminated (Due to slow/insufficient accrual)	National Cancer Institute, Bethesda, MD, USA
